# Morgan County Medical Society

**Published:** 1867-01

**Authors:** R. E. McVey, C. T. Wilbur

**Affiliations:** Pres’t.; Sec’y.


### MORGAN COUNTY MEDICAL SOCIETY.

---

Thursday, December 11th, 11 o'clock, A.M. Society met pursuant to adjournment.

Dr. Wagely was elected to preside, in the absence of the President and Vice-President.

The Secretary read the minutes of the last meeting, which were adopted.

The President called for the report of committees.

Committee on examination of credentials reported unanimously in favor of Dr. Dutton for membership, who was immediately elected by unanimous vote.

The committee on fee bill reported that they would be prepared to submit to the Society the result of their labors at the afternoon session.

Dr. Prince exhibited several varieties of instruments for producing spray for medication of the air-passages, and for deadening the sensibilities of parts in order to lessen or prevent the pain attendant upon surgical or dental operations. He went briefly into a history of the introduction of the method of inhaling medical substances blown off in spray, as first practiced in France and Germany, and then explained the instrument invented by Dr. Richardson, of London, for the production of insensibility of parts by blowing upon them a spray of ether or rhigoline, which is capable of freezing in a few seconds by the tendency of rapid evaporation to produce cold. He exhibited an ingenious modification of the apparatus devised by Dr. Black for the purpose of shielding the lips and tongue from the spray while it is being blown upon the gums preparatory to extracting teeth.

Dr. Prince referred to cases in which union by the first intention had occured under unfavorable circumstances after the application of the spray of ether to the cut surfaces. He thought this result was secured by the speedy arrest of the flow of blood from the minute vessels under the influence of cold.

Dr. Edgar confirmed this view, by selecting cases occuring in connection with the battles at Vicksburg, in which union by the first intention had been secured by the application of ice to the wounds immediately after the amputation and before the final dressing.

Dr. Black made some remarks explanatory of his employment of the spray in extracting teeth, thinking the instrument a valuable means of lessening or destroying sensibility. In some cases he had found patients unable to bear the sudden reduction of the temperature on account of the exposed and irritable condition of the nerve of the tooth. In some of these cases he had succeeded in employing the spray by first covering the tooth with wax.

On motion it was resolved that the thanks of the Society be tendered Dr. Black for his valuable contribution to dental surgery.

The subject of local anæsthesia being under consideration, Dr. Edgar related a case of anchylosis of the elbow-joint of a young lady, treated with chloroform and olive oil to the joint, enclosed in oil silk to prevent evaporation, by which means all sensation was removed, the adhesions were broken up and the joint restored.

Society adjourned to meet at 2 P.M.

At 2 o'clock P.M., the Society met. The Vice-President in the chair.

Dr. Warriner presented samples of his purified castor oil to the society for inspection and trial.

Committee on fee bill, Drs. Askew, Fisher, and Prince, submitted the following list of prices, for the establishment of a somewhat uniform rate of compensation for medical and surgical practice in the county:

For the purpose of a general guide in grading compensation for professional services, the following estimate is accepted by the members of the Morgan County Medical Society:

The higher charges for services of a surgical nature do not imply greater attainments than are required for skilful medical practice, but they are considered necessary in view of the less frequency of the cases and the expense necessarily incurred in providing instruments and apparatus.

Each member is still left to his full discretion to increase or diminish his own rate of compensation, in view of the pecuniary circumstances of his patients, or as a conscientious estimate of the value of his services, compared with those of members of greater or less attainments.

| | |
|---|---|
| Ordinary office advice, not consuming much time, and involving no unusual care in investigation, | $1 00 |
| Careful investigation in a physician's office or elsewhere, consuming considerable time, and | |

often requiring the introduction of a sound or catheter, the employment of chemical tests, the introduction of an exploring needle, the employment of a speculum, a stethescope, an ophthalmoscope, or a laryngoscope, by those skilled in these means of investigation,------- 5 00 to 25 00

Visit in town------------------------------- 1 50 to 3 00

Subsequent visits same day without special call, 1 00 to 2 00

Night Visit,------------------------------- 3 00 to 5 00

Extra patients in same family, each,----------- 1 00

Mileage—day,------------------------------- 1 00

" —night,------------------------------- 2 00

Obstetrics, uncomplicated, within three miles,--- 10 00 to 25 00

Delivery, by turning, forceps, or perforation,--- 25 00 to 50 00

Subsequent visits in town for the first three days, to be included in the charge, unless fever, inflammation, or other complication render unusual attention necessary.

Subsequent visits in the country, the same as in other cases.

Attendance on small-pox, per visit, (mileage extra),------------------------------- 5 00

Consultations (mileage extra),------------------- 5 00 to 10 00

Gonorrhœa and syphilis, in advance,------------ 10 00 to 50 00

Minor surgical operations, like opening abscesses, dressing bruised fingers, bleeding, cupping, the formation of issues, and the introduction of setons,------------------------------- 1 00 to 5 00

Dressing injuries of greater extent or danger, including fractures and dislocations, easily treated, and the ligation or acupression of arteries in wounds of little importance, the amputation of toes and fingers, circumcision, the removal of the tonsils—the uvula, etc.,---------------- 5 00 to 25 00

Dressing large or dangerous wounds, requiring the closure of important arteries to arrest hemorrhage, adjusting fractures and dislocations of greater magnitude or involving greater difficulties, the operation for hydrocele, for hair-lip, for strabismus, paracentesis, amputation of the breast, castration, the removal of tumors, not involving great difficulties, staphytorephy, laryngolemy, eridectomy, amputation, or extrac- of the eye, ---------------------------------- 25 00 to 100

Capital operations, including the larger amputations, resections, and exsections, and the removal of the paroted gland, trephining, ovariotemy, herinotomy, lithomy, the more difficult plastic operations, the reduction of dislocations which have resisted previous attempts, the adjustment of oblique fractures of the thigh and of those involving the neck of the femur, or of the knee-joint, compound and comminuted fractures of the larger bones and joints, extraction of cataract, and the formation of artifical pupil, ---------------------------------- 100 to 1,000

Subsequent attendance the same as in other cases.

The President declared any remarks on the subject to be in order.

Dr. Cassell thought a fee bill might be referred to as a general guide, but that great latitude must be allowed for varying circumstances, which the physican must respect.

Drs. Craig, Askew, Reed, Fisher, Johnson, and others offered remarks on the subject, when the list of prices, as submitted by the committee, was adopted by unanimous vote.

The Vice-President reported a novel and interesting case of menstruation and escape of liquor amnii during eight months of pregnancy.

Dr. Wagely moved the subject of puerperal fever be taken up.

Dr. Prince apologized for the delay of his paper on that subject, and promised it should be forthcoming at the next meeting.

Dr. Edgar called attention to the liability of error in practice from failure to diagnose correctly the pathological condition in each case of puerperal fever, *i.e.*, whether dependent on a true toxæmia, or on metritis, phlebitis, peritonitis or metroperitonitis. That the toxæmic condition being attended with an asthenic grade a fever indicated a supporting treatment while the purely inflammatory cases might be better treated by early free depletion and full exhibition of anodynes.

The further consideration of the subject was postponed until the next meeting.

On motion the Society adjourned to meet on the second Thursday of January, 1867, at 1 o'clock P.M.

R. E. McVEY, M.D., *Pres't.*

C. T. WILBUR, M.D., *Sec'y.*

---

